# Pulmonary Function Modulates Epigenetic Age in Subjects with Cystic Fibrosis

**DOI:** 10.3390/ijms26146614

**Published:** 2025-07-10

**Authors:** Alice Castaldo, Mariella Cuomo, Paola Iacotucci, Vincenzo Carnovale, Lorenzo Chiariotti, Giuseppe Castaldo, Monica Gelzo

**Affiliations:** 1 SC di Pneumologia e UTSIR, AORN Santobono-Pausilipon, 80129 Naples, Italy; alice.castaldo@unina.it; 2 Dipartimento di Scienze Mediche Traslazionali, Università di Napoli Federico II, 80131 Naples, Italy; vincenzo.carnovale@unina.it; 3 CEINGE-Biotecnologie Avanzate Franco Salvatore, 80145 Naples, Italy; mariella.cuomo@unina.it (M.C.); chiariot@unina.it (L.C.); monica.gelzo@unina.it (M.G.); 4 Dipartimento di Medicina Molecolare e Biotecnologie Mediche, Università di Napoli Federico II, 80131 Naples, Italy; 5 Dipartimento di Medicina Clinica e Chirurgia, Università di Napoli Federico II, 80131 Naples, Italy; paola.iacotucci@unina.it

**Keywords:** cystic fibrosis, epigenetic age, elexacaftor/tezacaftor/ivacaftor

## Abstract

Cystic fibrosis (CF) is the most common severe autosomal recessive disease among Caucasians. Modulators of cystic fibrosis transmembrane conductance regulator (CFTR) mutated protein significantly improved the outcome of subjects with CF. In the present study, we studied epigenetic age, applying the Horvath clock model, in 52 adult subjects with CF, all treated with elexacaftor/tezacaftor/ivacaftor (ETI). At baseline (T0), we found that half of the subjects have a significantly accelerated epigenetic age and a worse lung function, evaluated by forced expiratory volume in one second (FEV1). One year of ETI therapy (T1) impacted both the parameters, indicating that therapy with modulators must be started early, particularly in CF subjects with impaired lung function. The second group of CF subjects had an epigenetic age lower than the chronological one at T0 and lung function was better maintained. In these subjects, ETI therapy further improved lung function and tended to increase the epigenetic age, possibly improving metabolic functions and the general state of well-being. This also translates into an increase in the physical activities of a group of subjects who, before the therapy, had grown up under a glass bell. The analysis of epigenetic age may represent a potential biomarker to assess the individual outcome of the therapy in subjects with CF, although long-term studies need to evaluate it.

## 1. Introduction

Cystic fibrosis (CF) is the most common severe autosomal recessive disease among Caucasians. The altered amount or activity of the cystic fibrosis transmembrane conductance regulator protein (CFTR) impairs mucus hydration, resulting in a multisystem disease that includes pancreatic insufficiency (PI) with malabsorption and malnutrition, lung inflammation, and microbial colonization, leading to respiratory failure, liver disease, and CF-related diabetes (CFRD) [1]. Two thousand *CFTR* variants have a different impact on the protein and the study of their effect, also through ex vivo models [2,3], has expanded the number of patients accessing mutation-guided therapy with CFTR modulators [4,5]. Among these, the triple combination of elexacaftor/tezacaftor/ivacaftor (ETI) significantly improved the outcome of subjects with CF, particularly their pulmonary disease [5].

Several studies have shown that the severity of lung disease varies among CF patients and is influenced by genetic and non-hereditable factors, i.e., environmental factors. Genetic factors have been well studied, while the action mechanism of environmental factors on the CF clinical phenotype are still not well understood [6].

Recently, epigenetic modifications, including gene methylation, emerged as the link between environmental factors and phenotypic variability in CF [6], through the modulation of gene expression. Moreover, some factors typically associated with CF, such as diabetes and impaired lung function, can cause epigenetic age acceleration, as revealed by the methylation profiling of aging-related genes in non-CF subjects [7]. Thus, epigenetic age, referred as the measure of the biological age based on the DNA methylation levels at specific CpG sites, could contribute to assessing disease progression and the impact of mutation-directed therapies in subjects with CF.

## 2. Methods

We studied epigenetic age by analyzing DNA from the circulating cells [8] of all eligible CF adults for ETI treatment [5], enrolled from February 2021 to April 2023 and followed in our regional center, i.e., 52 subjects, median age 28.0 years, interquartile range: 22.3–36.5 years, 30 females (57.7%), at baseline (T0) and after one year (T1) of treatment with elexacaftor/tezacaftor/ivacaftor (ETI). The study was approved by the Ethics Committee of the University Federico II. Exclusion criteria included only refusal to participate in the study (no cases). The CFTR genotype was analyzed by gene sequencing [9].

All clinical data of subjects with CF were collected as in a previous study [10]. Respiratory function was assessed by spirometry examination. Forced expiratory volume in one second (FEV1) was chosen as the best spirometry value in CF patients for its clinical relevance, its association with morbidity and mortality, its role in epidemiologic studies and clinical trials, assessing the disease progression and the efficacy of new therapies [11].

The Infinium MethylationEPIC array v2.0 BeadChip (Illumina, San Diego, CA, USA) was used to analyze DNA methylation status from blood cells at 935,000 CpG sites. Raw intensity data (IDAT format) were preprocessed in the R statistical environment (version 4.4.3) using the RnBeads pipeline analysis package (version 2.0) [12]. Epigenetic age was calculated using the R package dnaMethyAge (version 0.2.0) and applying the Horvath clock model as biomarker of aging based on mathematical models that elaborate DNA methylation patterns to estimate a subject’s biological age [8]. Specifically, we selected the Horvath Skin & Blood clock for estimating epigenetic age because it has been shown to perform with high accuracy in blood samples, and importantly, it exhibits strong concordance between blood and other somatic tissues [13,14]. Detailed protocols are available upon request.

For each subject included in the study, we calculated the epigenetic age ratio (ER) as follows:

ER = [(epigenetic age − chronological age)/chronological age] × 100(1)

Parametric (FEV1%) and non-parametric (all other variables) data are reported as mean (standard deviation) and median (interquartile range), respectively. Comparisons between negative and positive ER were performed by Mann–Whitney U test, while comparisons between T0 and T1 parameters were performed by Wilcoxon test. We applied stratified statistics controlling for confounding factors. However, no significant differences in potential confounders, such as gender, chronological age and BMI, have been observed. Correlations between variables were evaluated using Spearman’s correlation analysis. Linear regression analysis was used to assess the effect of FEV1 (independent variable) on ER (dependent variable) by the stepwise method. Statistical analyses were performed by SPSS (version 29, IBM SPSS Statistics, Armonk, NY, USA).

## 3. Results and Discussion

As shown in Table 1, the ER at T0 resulted negative in 26/52 subjects (50%),with epigenetic age being significantly lower than the chronological age. In the remaining 26 (50%) the ratio was positive, with the epigenetic age being significantly higher than the chronological one. Interestingly, in both subgroups the gender distribution was the same, thus excluding the impact of gender on the epigenetic age, and no significant difference in the chronological age was observed between the two subgroups. In subjects with a negative ratio at T0, the treatment increased the ratio (although not significantly), while, in subjects with a positive ratio at T0, the ratio significantly increased after one year of treatment, indicating the positive impact of the therapy on epigenetic age. Therefore, we compared clinical data between the two subgroups of CF subjects both at T0 and T1.

Both body mass index and sweat chloride (SC) were not significantly different between the two subgroups neither at T0 nor at T1, and in both subgroups the two parameters were significantly improved after one year of therapy, in agreement with previous results [15,16]. The FEV1 value was significantly lower in the subgroup with a positive ratio, both at T0 and T1, indicating that lung function has a significant impact on epigenetic age in CF subjects, as previously described in non-CF populations [7]. Interestingly, the ETI therapy significantly improved FEV1 both in the ER-negative and ER-positive subgroups of CF subjects, likely contributing to the reduction in epigenetic age in this latter subgroup.

**Figure 1:**
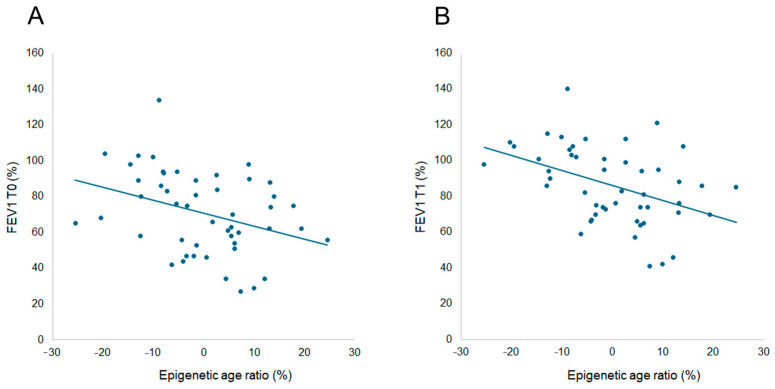
Spearman correlation analysis between epigenetic age ratio and FEV1% in 52 subjects with cystic fibrosis at baseline (T0, panel (**A**)) and after one year of therapy with elexacaftor/tezacaftor/ivacaftor (T1, panel (**B**)). Epigenetic age ratio: (epigenetic age − chronological age)/chronological age × 100.

In addition, ER was inversely correlated with FEV1 both at T0 (r_s_ = −0.377, *p* = 0.008, Figure 1A) and T1 (r_s_ = −0.438, *p* = 0.002, Figure 1B). Thus, we assessed the effect of FEV1 on ER by linear regression analysis and we found that ER was negatively related to FEV1 both at T0 (slope = −0.348; *p* = 0.014) and T1 (slope = −0.423; *p* = 0.002).

No correlations were found between the ER and SC, and no differences in the ER were found at both T0 and T1 between subjects with and without PI, CF hepatobiliary disease [17], CFRD, and *P. aeruginosa* colonization, except between subjects with and without PI at T1 (Table S1).

This study showed the existence of two groups of subjects with CF (although the study is limited to one year of follow up with ETI therapy). Half of the subjects had a significantly accelerated epigenetic age and a worse lung function. The therapy with ETI impacted on both the two parameters, indicating that therapy with modulators must be started early, particularly in CF subjects with impaired lung function. The second group of CF subjects had an epigenetic age lower than the chronological one and the lung function was better maintained. In these subjects, ETI therapy further improved lung function and tended to increase the epigenetic age, possibly improving metabolic functions and the general state of well-being. This translates also into an increase in the physical activities [16,18] of a group of subjects who, before the therapy, had grown up under a glass bell. This analysis of epigenetic age may represent a potential biomarker to assess the individual outcome of the therapy in subjects with CF, although long-term studies need to evaluate it.

## Figures and Tables

**Table 1:** Comparison of epigenetic age ratio (ER) and demographic and clinical parameters between CF subjects with negative and positive ER at baseline (T0) and after one year (T1) of therapy with ETI.

Parameters	Negative ER at T0 (n = 26)	Positive ER at T0 (n = 26)	*p* Value
Females, n (%)	15 (57.7%)	15 (57.7%)	-
Chronological age T0 (years)	25.5 (21.0, 37.5)	29.5 (24.5, 36.0)	0.256
Epigenetic age T0 (years)	22.6 (18.1, 36.7)	33.5 (27.4, 41.3)	**0.006**
*p* value	**<0.001**	**<0.001**	
Epigenetic age ratio T0	−8.0 (−12.9, −3.9)	8.1 (5.3, 13.5)	**<0.001**
Epigenetic age ratio T1	−5.3 (−15.2, 0.4)	3.2 (−1.3, 9.9)	**<0.001**
*p* value	0.238	**0.001**	
BMI T0 (kg/m^2^)	22.5 (21.4, 25.9)	22.1 (20.1, 23.4)	0.080
BMI T1 (kg/m^2^)	23.3 (22.0, 26.3)	23.7 (21.7, 25.2)	0.596
*p* value	**0.007**	**<0.001**	
Sweat chloride T0 (mmol/L)	66.0 (41.0, 78.5)	69.0 (60.0, 80.5)	0.317
Sweat chloride T1 (mmol/L)	28.0 (20.0, 30.5)	22.0 (12.2, 34.8)	0.246
*p* value	**<0.001**	**<0.001**	
FEV1 T0 (%)	77.2 (23.3)	63.8 (19.8)	**0.030**
FEV1 T1 (%)	92.2 (20.9)	78.7 (20.3)	**0.021**
*p* value	**<0.001**	**<0.001**	

Epigenetic age ratio: (epigenetic age − chronological age)/chronological age × 100. ETI: elexacaftor/tezacaftor/ivacaftor; BMI: body mass index; FEV1: forced expiratory volume in 1 s. Significant *p*-values are reported in bold.

## Data Availability

The raw data supporting the conclusions of this article will be made available by the authors on request, as the data are part of an ongoing study.

## Supplementary Materials

The following supporting information can be downloaded at: https://www.mdpi.com/article/10.3390/ijms26146614/s1.

## Abbreviations

The following abbreviations are used in this manuscript:

CF	Cystic fibrosis
CFTR	Cystic fibrosis transmembrane conductance regulator
PI	Pancreatic insufficiency
CFRD	CF-related diabetes
ETI	Elexacaftor/tezacaftor/ivacaftor
ER	Epigenetic age ratio
CFHBI	CF hepatobiliary involvement
BMI	Body mass index
SC	Sweat chloride
FEV1	Forced expiratory volume in 1 s
