# Supplementary material for: Pulmonary Function Modulates Epigenetic Age in Subjects with Cystic Fibrosis
Source: Int J Mol Sci. 2025 Jul 10;26(14):6614. doi: 10.3390/ijms26146614 (PMC12295467; doi:10.3390/ijms26146614)
Supplement: Supplementary file 1 [file ijms-26-06614-s001.zip › ijms-3711901-supplementary.pdf]

**Table S1.** Comparison of epigenetic age ratio between CF subjects with and without PI, CFHBI, CFRD, and PA colonization at baseline (T0) and after 1 year (T1) therapy with elexacaftor/tezacaftor/ivacaftor (ETI).

| | Baseline | | | After 1 year of ETI | | |
|---|---|---|---|---|---|---|
| | PI (n=48) | no PI (n=4) | p value | PI (n=48) | no PI (n=4) | p value |
| Epigenetic age ratio (%) | 2.2 (-7.7, 9.1) | -6.5 (-17.0, -3.5) | 0.090 | 0.4 (-4.4, 5.0) | -7.7 (-12.6, -6.4) | **0.031** |
| | CFHBI (n=16) | no CFHBI (n=36) | p value | CFHBI (n=16) | no CFHBI (n=36) | p value |
| Epigenetic age ratio (%) | 1.2 (-11.0, 12.2) | -1.5 (-8.0, 7.3) | 0.905 | 2.7 (-10.9, 7.0) | -1.3 (-5.8, 3.1) | 0.372 |
| | CFRD (n=21) | no CFRD (n=31) | p value | CFRD (n=21) | no CFRD (n=31) | p value |
| Epigenetic age ratio (%) | 2.6 (-10.4, 6.8) | -1.9 (-7.9, 9.1) | 0.904 | 2.6 (-5.4, 5.3) | -1.6 (-7.1, 3.2) | 0.520 |
| | PA (n=31) | no PA (n=21) | p value | PA (n=31) | no PA (n=21) | p value |
| Epigenetic age ratio (%) | 4.5 (-6.3, 8.9) | -3.2 (-10.8, 7.7) | 0.346 | 0.1 (-4.1, 5.2) | -2.4 (-13.3, 3.3) | 0.318 |

PI: pancreatic insufficiency; CFHBI: cystic fibrosis-related hepatobiliary involvement; CFRD: cystic fibrosis-related diabetes; PA: *Pseudomonas aeruginosa* colonization. Epigenetic age ratio: (epigenetic age – chronological age)/chronological age * 100.
